# Clinical characteristics of patients with GFAP-IgG coexisting with AQP4-IgG or MOG-IgG

**DOI:** 10.3389/fimmu.2025.1610486

**Published:** 2025-07-24

**Authors:** Qingchen Li, Xinyun Chen

**Affiliations:** ^1^ Department of Neurology, the First Affiliated Hospital of Zhengzhou University, Zhengzhou, China; ^2^ Department of Neurology, the Third People’s Hospital of Zhengzhou, Zhengzhou, China

**Keywords:** glial fibrillary acidic protein, aquaporin-4, myelin oligodendrocyte glycoprotein, autoimmunity, overlapping

## Abstract

**Background:**

Glial fibrillary acidic protein–immunoglobulin G (GFAP-IgG) can coexist with aquaporin-4–IgG (AQP4-IgG) or myelin oligodendrocyte glycoprotein–IgG (MOG-IgG). We aimed to investigate the clinical characteristics of patients with GFAP-IgG coexisting with AQP4-IgG or MOG-IgG.

**Methods:**

We retrospectively collected data from 81 GFAP-IgG-positive patients and described and compared the clinical characteristics of those with GFAP-IgG coexisting with AQP4-IgG or MOG-IgG.

**Results:**

(1) Among the 81 GFAP-IgG-positive patients, nine (11.1%) were positive for AQP4-IgG and seven (8.6%) were positive for MOG-IgG. The clinical manifestations of overlapping syndromes were diverse; all patients met the clinical phenotype of autoimmune GFAP astrocytopathy (A-GFAP-A) and also fulfilled the diagnostic criteria for neuromyelitis optica spectrum disorders or MOG antibody-associated disorders. Compared with the GFAP-AQP4 overlapping syndrome, the GFAP-MOG overlapping syndrome had a higher frequency of seizures (57.1% vs. 0, *p* = 0.019). (2) Compared with the nonoverlapping syndrome group, the overlapping syndrome group had more women (68.6% vs. 32.3%, *p* = 0.008), a higher incidence of optic neuritis (ON) (43.8% vs. 4.6%, *p* < 0.001), lower CSF white blood cell counts (median: 30 cells/mm^3^ vs. 94 cells/mm^3^, *p* = 0.001) and protein levels (median: 0.375 g/L vs. 0.78 g/L, *p* < 0.001), and a higher proportion of patients receiving long-term immunotherapy (68.8% vs.13.8%, *p* < 0.001).

**Conclusions:**

Among patients with A-GFAP-A, 20% had concurrent AQP4-IgG or MOG-IgG, exhibiting distinct clinical features that suggest a different disease phenotype driven by overlapping autoimmune mechanisms.

## 1 Introduction

Autoimmune glial fibrillary acidic protein astrocytopathy (A-GFAP-A) is an autoimmune inflammatory disorder of the central nervous system (CNS) that affects the brain, meninges, spinal cord, and optic nerve. The primary clinical manifestations include corticosteroid-responsive encephalitis or meningoencephalitis, with or without myelitis. An immunoglobulin G (IgG) autoantibody that selectively targets GFAP in astrocytes has been identified as a highly specific diagnostic biomarker for this disease (1, 2). Neuromyelitis optica spectrum disorders (NMOSD) and myelin oligodendrocyte glycoprotein (MOG) antibody-associated disorders (MOGAD) are two immune-mediated inflammatory demyelinating diseases of the CNS. Aquaporin-4 (AQP4) antibody and MOG antibody serve as specific biomarkers for the diagnosis of these two diseases, respectively (3, 4).

The clinical manifestations of A-GFAP-A, NMOSD, and MOGAD exhibit significant overlap, and in some cases, GFAP-IgG may co-occur with AQP4-IgG or MOG-IgG (5–11). However, most previous studies were case reports, and no study has systematically compared the frequency, clinical features, and auxiliary examinations of these overlapping syndromes. Here, we analyzed the clinical characteristics of patients with GFAP-IgG coexisting with either AQP4-IgG or MOG-IgG, aiming to enhance our understanding of this phenomenon.

## 2 Materials and methods

### 2.1 Patients

The study was approved by the ethics committee of the First Affiliated Hospital of Zhengzhou University (2024-KY-2298).

In this study, we identified 81 GFAP-IgG-positive patients who were admitted to the First Affiliated Hospital of Zhengzhou University, China, from February 2020 to October 2024. The inclusion criteria included the following: (1) CSF and/or serum testing positive for GFAP-IgG by cell-based assays (CBA); (2) presentation with one or more clinical syndromes, including encephalitis, meningitis, myelitis, and optic neuritis (ON); (3) testing for AQP4-IgG and MOG-IgG by CBA; and (4) availability of clinical data. The exclusion criteria included other diseases, such as brain tumors, traumatic brain injury, Alzheimer’s disease, and toxic or metabolic CNS disorders.

### 2.2 Study design

We retrospectively analyzed the clinical data of these patients. Information was collected through medical records, telephone interviews, and outpatient follow-ups. The modified Rankin Scale (mRS) was used to assess disease severity and clinical outcomes at the last follow-up; an mRS score < 3 was considered a good outcome. Follow-up duration was defined as the time from the initial evaluation at disease onset to the last follow-up. Relapses were defined as the development of new neurological symptoms lasting at least 24 h, occurring 1 month after clinical improvement or stabilization.

All CSF and serum samples were collected during the early active disease stage. GFAP antibodies were detected using an indirect immunofluorescence CBA method, employing human embryonic kidney (HEK) 293 cells transfected with GFAP expression plasmids (Shanghai Genechem Co.,Ltd, Shanghai, China). Antibodies to AQP4 and MOG were detected using the fixed CBA. Among the 81 GFAP-IgG-positive patients, nine were positive for AQP4-IgG and seven were positive for MOG-IgG. We characterized the clinical features of these overlapping syndromes and compared them with those of patients without coexisting AQP4-IgG or MOG-IgG.

### 2.3 Statistical analysis

Statistical analyses were performed using SPSS version 26.0. Continuous variables were expressed as mean ± standard deviation (SD) or median (range), and categorical variables as counts and proportions. Continuous variables were compared using the Mann–Whitney *U* test or *t*-test, and categorical variables using the Chi-square test or Fisher’s exact test. A *p*-value < 0.05 was considered statistically significant.

## 3 Results

### 3.1 Case series study

#### 3.1.1 Demographics and clinical manifestations

Among the 81 GFAP-IgG-positive patients, nine (11.1%) were positive for AQP4-IgG (patients 1–9; one man, eight women), and seven (8.6%) were positive for MOG-IgG (patients 10–16; four men, three women). The overlapping syndromes were defined as follows: the coexistence of GFAP-IgG and AQP4-IgG was categorized as the GFAP-AQP4 group, while the coexistence of GFAP-IgG and MOG-IgG was categorized as the GFAP-MOG group. The clinical data of patients with these two overlapping syndromes are summarized in **Tables 1**, **2**.

**Table 1:** Clinical data of patients with the GFAP-AQP4 overlapping syndrome.

No./sex/age (year)	GFAP-IgG serum/CSF	AQP4-IgG serum/CSF	Clinical manifestations	Phenotypes/core clinical characteristics of NMOSD	MRI abnormality	CSF findings: WBC (/mm^3^), protein (g/L)	Therapy	Duration of follow-up (months)	mRS (max/last follow-up)
1/F/32	−/+	−/+	Fever, headache, psychiatric symptoms, hypersomnia	Encephalitis/acute diencephalic syndrome	Bilateral thalamus, fornix, periventricular region, optic chiasm	WBC: 30; protein: 0.297; OCBs: +	IVMP, IVIG, AZA	3	5/1
2/F/14	+/+	+/+	Fever, headache, diplopia, hypersomnia, visual impairment	Encephalomyelitis/acute myelitis, acute diencephalic syndrome, ON	Periventricular region, corpus callosum, inferior colliculus, thalamus, basal ganglia, midbrain, optic chiasm, C2–3, T4–6, T8	WBC: 39; protein: 0.36; OCBs: −	IVMP, AZA	4	4/0
3/F/48	−/+	+/+	Paralysis, limb numbness, dysuria	Myelitis/acute myelitis	Medulla oblongata, C1–T9	WBC: 2; protein: 0.405; OCBs: +	IVMP, RTX, tocilizumab	9	4/3
4/F/44	−/+	+/+	Paralysis, limb numbness	Myelitis/acute myelitis	C1–T1	WBC: 28; protein: 0.425; OCBs: −	IVMP, IVIG, MMF	36	3/0
5/F/12	−/+	+/+	Fever, intractable nausea and hiccups, diplopia, ear pain, facial paralysis, ataxia, visual impairment	Encephalomyelitis/acute myelitis, APS, acute brainstem syndrome, ON	Periventricular region, pons, medulla oblongata, optic nerve, C1–T1, T7, T10–11	WBC: 31; protein: 0.443; OCBs: +	IVMP, IVIG, MMF, RTX	17	4/3
6/M/27	+/−	+/−	Fever, cognitive impairment, neck stiffness, paralysis, dysuria	Meningoencephalomyelitis/acute myelitis, symptomatic cerebral syndrome	Bilateral frontal lobe, periventricular region, left thalamus, pons, C2–7, T2–12, leptomeningeal enhancement	WBC: 38; protein: 0.406; OCBs: −	IVMP	7	4/1
7/F/34	−/+	−/+	Fever, intractable nausea and hiccups, diplopia	Encephalomyelitis/acute myelitis, acute brainstem syndrome, APS	Left frontotemporal lobe, medulla oblongata, T2–3, T12–L1	WBC: 87; protein: 0.212; OCBs: +	IVMP, MMF	11	3/1
8/F/35	−/+	+/+	Paralysis, limb numbness, dysuria, neck stiffness	Meningoencephalomyelitis/acute myelitis, symptomatic cerebral syndrome	Medulla oblongata, C1–6, T2–6, leptomeningeal enhancement	WBC: 2; protein: 0.203; OCBs: −	IVMP, TAC	34	3/1
9/F/55	−/+	+/+	Nausea and vomiting, facial pain, dysphagia, limb numbness	Encephalitis/acute brainstem syndrome, symptomatic cerebral syndrome	Bilateral frontal lobe, parietal lobe, and periventricular region	WBC: 24; protein: 0.368; OCBs: −	IVMP	5	3/3

*APS*, area postrema syndrome; *AQP4*, aquaporin-4; *AZA*, azathioprine; *CSF*, cerebrospinal fluid; *F*, female; *GFAP*, glial fibrillary acidic protein; *IgG*, immunoglobulin G; *IVIG*, intravenous immunoglobulins; *IVMP*, intravenous methylprednisolone; *M*, male; *Max*, maximum; *MMF*, mycophenolate mofetil; *MRI*, magnetic resonance imaging; *mRS*: modified Rankin Scale; *NMOSD*, neuromyelitis optica spectrum disorders; *OCBs*, oligoclonal bands; *ON*, optic neuritis; *RTX*, rituximab; *TAC*, tacrolimus; *WBC*, white blood cell.

**Table 2:** Clinical data of GFAP-MOG overlapping syndrome.

No./sex/age (year)	GFAP-IgG serum/CSF	MOG-IgG serum/CSF	Clinical manifestations	Phenotypes	MRI abnormality	CSF findings: WBC (/mm^3^), protein (g/L)	Therapy	Duration of follow-up (months)	mRS (max/last follow-up)
10/F/11	+/+	+/+	Psychiatric symptoms, seizure, hypersomnia, neck stiffness, paralysis, dysuria	Meningoencephalomyelitis	C2–6, T9–12	WBC: 18; protein: 0.262; OCBs: +	IVMP. IVIG, PLEX	6	5/0
11/M/36	−/+	+/+	Fever, headache, paralysis, limb numbness, dysuria, visual impairment	Encephalomyelitis, ON	Left frontal cortex/subcortex, thalamus, periventricular region, cerebral peduncle, pons, bilateral cerebellum, optic nerve, C1–T3	WBC: 38; protein: 0.389; OCBs: −	IVMP, IVIG, MMF	13	4/0
12/M/5	−/+	+/+	Headache, diplopia, hypersomnia, neck stiffness	Meningoencephalomyelitis	Right frontal lobe, bilateral parietotemporal cortex/subcortex, basal ganglia, thalamus, midbrain, C5–T12, C3–6	WBC: 16; protein: 0.294; OCBs: −	IVMP, IVIG	3	4/0
13/F/3	+/−	+/+	Fever, headache, hypersomnia, visual impairment	Encephalomyelitis, ON	Bilateral frontal, parietal, and temporal lobes; corpus callosum; basal ganglia; pons; left cerebellum; optic nerve; C4–T8	WBC: 22; protein: 0.407; OCBs: −	IVMP, IVIG, MMF, RTX, tocilizumab	34	3/0
14/M/52	+/+	+/−	Paralysis, limb numbness, seizure, cognitive impairment, neck stiffness, dysuria, respiratory failure, visual impairment	Meningoencephalitis, ON	Bilateral frontal and parietal lobes, bilateral periventricular region, bilateral basal ganglia	WBC: 11; protein: 0.872; OCBs: −	IVMP, IVIG	12	5/5
15/F/26	+/+	+/−	Fever, headache, nausea and vomiting, seizure, diplopia, dysuria, visual impairment	Meningoencephalitis, ON	Cerebral cortex, bilateral cerebellum, thalamus, basal ganglia, brainstem, leptomeningeal enhancement	WBC: 38; protein: 0.36; OCBs: −	IVMP, MMF	4	3/0
16/M/19	−/+	+/+	Headache, seizure, psychiatric symptoms, hypersomnia, visual impairment	Encephalitis, ON	Right frontal and parietal cortex	WBC: 44; protein: 0.381; OCBs: +	IVMP, IVIG, MMF	9	5/0

*CSF*, cerebrospinal fluid; *F*, female; *GFAP*, glial fibrillary acidic protein; *IgG*, immunoglobulin G; *IVIG*, intravenous immunoglobulins; *IVMP*, intravenous methylprednisolone; *M*, male; *Max*, maximum; *MMF*, mycophenolate mofetil; *MOG*, myelin oligodendrocyte glycoprotein; *MRI*, magnetic resonance imaging; *mRS*: modified Rankin Scale; *OCBs*, oligoclonal bands; *ON*, optic neuritis; *PLEX*, plasma exchange; *RTX*, rituximab; *WBC*, white blood cell.

The median age at onset was 34 years (range: 12–55 years) in the GFAP-AQP4 group and 19 years (range: 3–52 years) in the GFAP-MOG group. Patient 5 developed area postrema syndrome (APS) 40 days earlier, and patient 11 developed myelitis and ON 16 months earlier. However, antibodies against GFAP, AQP4, and MOG were not detected at that time. Upon experiencing new neurological events, both patients underwent antibody testing and were subsequently diagnosed. Tumor screening was performed in all patients, and elevated tumor markers were detected in three cases (CA72–4 in patients 5 and 16; CA19–9 in patient 15). However, no concurrent tumors were identified in the overlapping patients.

The clinical phenotypes in the GFAP-AQP4 group included encephalomyelitis (*n* = 3), meningoencephalomyelitis (*n* = 2), encephalitis (*n* = 2), and myelitis (*n* = 2), with two patients also presenting with ON. These syndromes fulfilled the core clinical characteristics of NMOSD, including acute myelitis (*n* = 7), acute brainstem syndrome (*n* = 3), symptomatic cerebral syndrome (*n* = 3), APS (*n* = 2), ON (*n* = 2), and acute diencephalic syndrome (*n* = 2). The clinical phenotypes in the GFAP-MOG group included meningoencephalomyelitis (*n* = 2), encephalomyelitis (*n* = 2), meningoencephalitis (*n* = 2), and encephalitis (*n* = 1); notably, five patients with ON. Compared with the patients in the GFAP-AQP4 group, those in the GFAP-MOG group had more seizures (57.1% vs. 0%, *p* = 0.019) (**Table 3**).

**Table 3:** Comparison of clinical characteristics among all groups.

Characteristic	GFAP-AQP4 group (n = 9)	GFAP-MOG group (n = 7)	Overlapping syndrome (n = 16)	Nonoverlapping syndrome (n = 65)	P1	P2	P3	P4
Female, (n(%)	8(88.9)	3(42.9)	11(68.6)	21(32.3)	0.106	0.004^*^	0.888	0.008^*^
Age at onset median(range), y	34(12, 55)	19(3,52)	30(3,55)	42(3,71)	0.153	0.661	0.075	0.154
Involved site, (n(%)								
Brain	7(77.8)	7(100)	14(87.5)	60(92.3)	0.475	0.200	1.000	0.907
Spinal cord	8(88.9)	4(57.1)	12(75)	42(64.6)	0.262	0.281	1.000	0.430
Optic nerve^a^	2(22.2)	5(71.4)	7(43.8)	3(4.6)	0.126	0.109	0.000^*^	0.000^*^
Unilateral optic nerve	0	0	0	2				
Bilateral optic nerve	2	5	7	1				
Seizure, (n(%)	0	4(57.1)	4(25)	10(15.4)	0.019^*^	0.456	0.032^*^	0.588
MRI findings, n(%)								
Abnormal brain MRI	8(88.9)	6(85.7)	14(87.5)	51(78.5)	1.000	0.774	1.000	0.643
Cortex	3(33.3)	6(85.7)	9(56.3)	23(35.4)	0.060	1.000	0.030^*^	0.126
Corpus callosum	1(11.1)	1(14.3)	2(12.5)	15(23.1)	1.000	0.700	0.958	0.557
Periventricular region	5(55.6)	2(28.6)	7(43.8)	22(33.8)	0.358	0.369	1.000	0.459
Thalamus	3(33.3)	3(42.9)	6(37.5)	20(30.8)	1.000	1.000	0.822	0.605
Basal ganglia	1(11.1)	4(57.1)	5(31.3)	22(33.8)	0.106	0.319	0.421	0.844
Cerebellum	0	3(42.9)	3(18.8)	15(23.1)	0.063	0.241	0.491	0.970
Brainstem	6(66.7)	4(57.1)	10(62.5)	23(35.4)	1.000	0.151	0.472	0.048^*^
Linear radial perivascular enhancement	0	0	0	5(7.7)	–	1.000	1.000	0.577
Abnormal spinal cord MRI	7(77.8)	4(57.1)	11(68.8)	41(63.1)	0.734	0.622	1.000	0.672
Cervical cord	6(66.7)	4(57.1)	10(62.5)	35(53.8)	1.000	0.713	1.000	0.533
Thoracic cord	7(77.8)	4(57.1)	11(68.8)	34(52.3)	0.596	0.279	1.000	0.236
Lumbar cord	1(11.1)	0	1(6.3)	5(7.7)	1.000	1.000	1.000	1.000
LETM	6(66.7)	4(57.1)	10(62.5)	31(47.7)	1.000	0.477	0.938	0.289
Leptomeningeal enhancement	2(22.2)	1(14.3)	3(18.8)	21(32.3)	1.000	0.684	0.482	0.202
CSF findings								
WBC, [(/mm^3^, median(range)]	31(2,87)	22(11,44)	30(2,87)	94(2,1,090)	0.816	0.011^*^	0.020^*^	0.001^*^
Protein, [(g/L, median(range)]	0.368(0.203,0.443)	0.381(0.262,0.872)	0.375(0.203,0.872)	0.780(0.228,4.433)	0.643	0.000^*^	0.004^*^	0.000^*^
Immunotherapy, (n(%)								
IVMP	9(100)	7(100)	16(100)	63(96.9)	–	1.000	1.000	1.000
IVIG	3(33.3)	6(85.7)	9(56.3)	30(46.2)	0.060	0.713	0.112	0.469
Plasma exchange	0	1(14.3)	1(6.3)	4(6.2)	0.438	1.000	0.410	1.000
Long-term immunotherapy	7(77.8)	4(57.1)	11(68.8)	9(13.8)	0.596	0.000^*^	0.021^*^	0.000^*^
Outcomes								
Follow-up(median(range), m	9(3,36)	9(3,34)	9(3,36)	9(3,46)	0.791	0.894	0.848	0.981
Good outcome, (n(%)	6(66.7)	6(85.7)	12(75)	49(75.4)	0.585	0.878	0.886	1.000
Polyphasic course, (n(%)	2(22.2)	2(28.6)	4(25)	5(7.7)	1.000	0.200	0.135	0.126

P1, GFAP-AQP4 group compared with GFAP-MOG group; P2, GFAP-AQP4 group compared with nonoverlapping syndrome; P3, GFAP-MOG group compared with nonoverlapping syndrome; P4, overlapping syndrome compared with nonoverlapping syndrome; *AQP4*, aquaporin-4; *CSF*, cerebrospinal ﬂuid; *GFAP*, glial fibrillary acidic protein; *IVIG*, intravenous immunoglobulin; *IVMP*, intravenous methylprednisolone; *LETM*, longitudinally extensive transverse myelitis; *MRI*, magnetic resonance imaging; *MOG*, myelin oligodendrocyte glycoprotein; *MRI*, magnetic resonance imaging; *nonoverlapping syndrome*, GFAP-IgG+/AQP4-IgG−/MOG-IgG−; *WBC*, white blood cell counts. ^*^With statistical significance. ^a^Visual field defect, vision loss, blurred vision, optic disk edema, and abnormal visual evoked potentials.

#### 3.1.2 MRI and CSF findings

**Figure 1:**
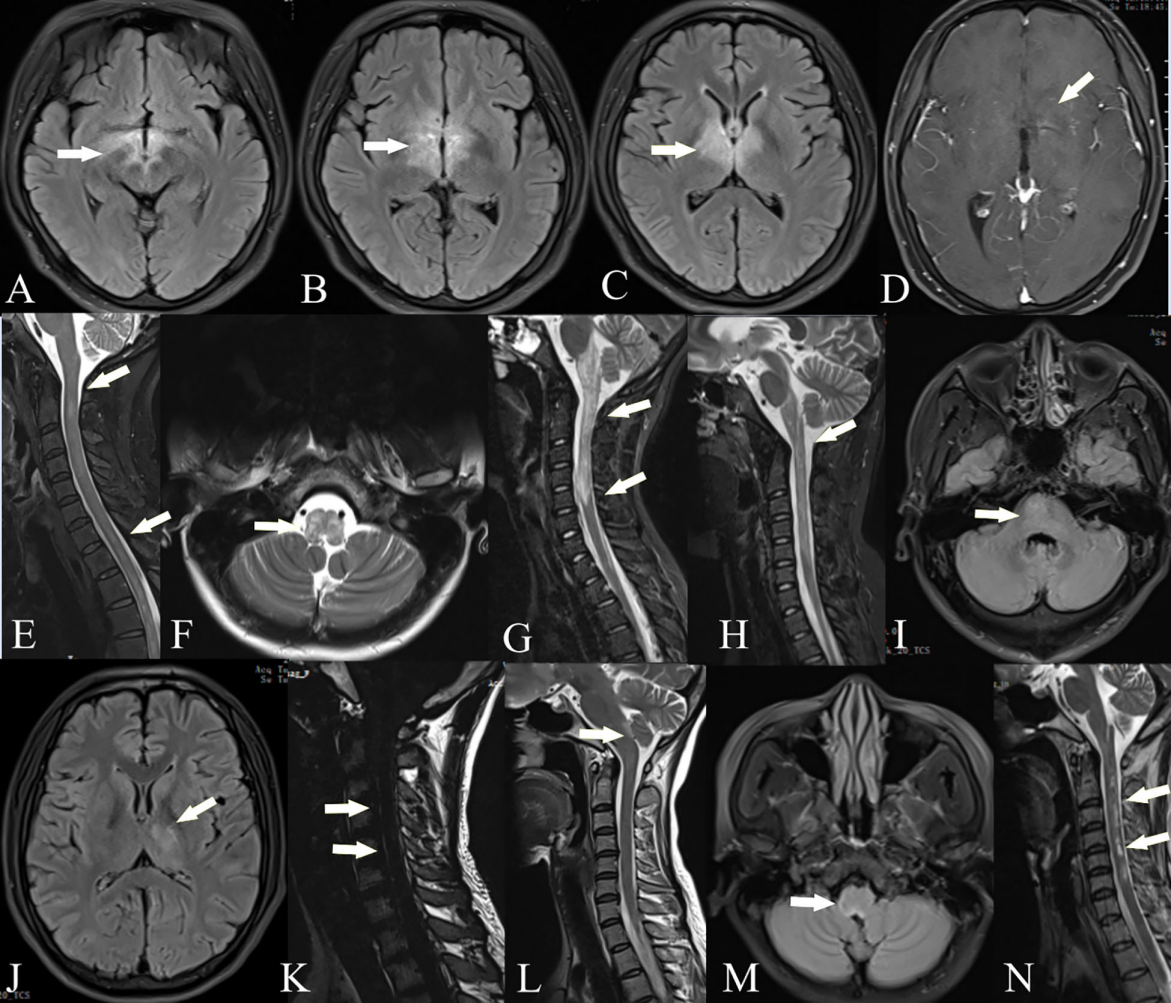
Brain and spinal cord MRI of GFAP-AQP4 overlapping syndrome. Patient 1: T2-FLAIR imaging showed lesions in the thalamus, fornix, optic chiasm, and around the third ventricle **(A–C)**. Patient 2: Contrast-enhanced T1-weighted imaging showed spotty enhancement in the corpus callosum and around the third ventricle **(D)**. Patient 3: T2-weighted imaging showed lesions in the spinal cord **(E)**. Patient 5: T2-weighted imaging showed lesions in the medulla oblongata **(F)** and spinal cord **(G)**; after treatment, the lesions were significantly reduced **(H)**. Patient 6: T2-FLAIR imaging showed lesions in the paraventricular region, thalamus, and pons **(I, J)**, with leptomeningeal enhancement in the cervical cord **(K)**. Patient 7: T2-FLAIR imaging showed lesions in the area postrema **(L, M)**. Patient 8: T2-weighted imaging showed lesions in the spinal cord **(N)**.

**Figure 2:**
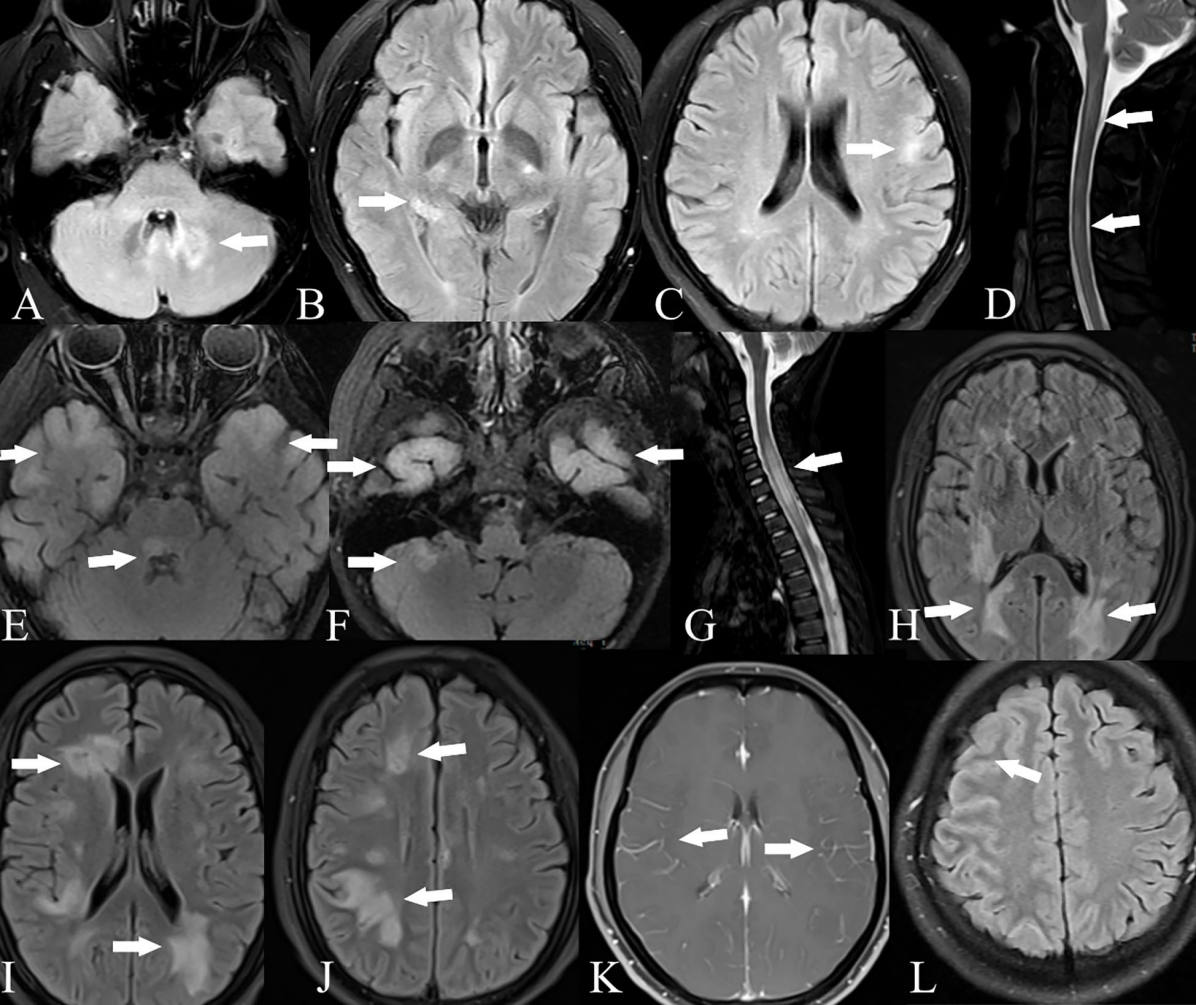
Brain and spinal cord MRI of GFAP-MOG overlapping syndrome. Patient 11: T2-FLAIR imaging showed lesions in the cerebellum **(A)**, thalamus **(B)**, and cortex **(C)**; T2-weighted imaging showed lesions in the spinal cord **(D)**. Patient 13: T2-FLAIR imaging showed lesions in the bilateral cortex, pons, and cerebellum **(E, F)**; T2-weighted imaging showed lesions in the spinal cord **(G)**. Patient 14: T2-FLAIR imaging showed lesions in the bilateral cortex, periventricular region, and basal ganglia **(H–J)**. Patient 15: Contrast-enhanced T1-weighted imaging showed leptomeningeal enhancement **(K)**. Patient 16: T2-FLAIR imaging showed lesions in the right frontal and parietal cortex **(L)**.

Brain and spinal cord MRIs were performed in all patients. Brain lesions were observed in eight patients in the GFAP-AQP4 group and six patients in the GFAP-MOG group. In the GFAP-AQP4 group, common lesions were observed in the brainstem (*n* = 6), periventricular region (*n* = 5), cortex (*n* = 3), and thalamus (*n* = 3). In contrast, the GFAP-MOG group exhibited common lesions in the cortex (*n* = 6), basal ganglia (*n* = 4), brainstem (*n* = 4), thalamus (*n* = 3), and cerebellum (*n* = 3). Leptomeningeal enhancement was observed in two patients (patient 8 in the brainstem meninges and patient 15 in the meninges of the cerebellar and cerebral hemispheres). However, no patients exhibited periventricular radial linear enhancement. Spinal MRI revealed that six patients in the GFAP-AQP4 group and four patients in the GFAP-MOG group had longitudinally extensive spinal cord lesions, primarily located in the cervicothoracic cord. Patient 7 had spinal lesions involving no more than three vertebral segments, while patient 4 had spinal meningeal enhancement. MRI characteristics are summarized in **Tables 1**, **2** and **Figures 1**, **2**.

CSF analysis revealed pleocytosis (> 5 cells/mm^3^) in seven patients from both the GFAP-AQP4 and GFAP-MOG groups. Increased protein levels (> 0.45 g/L) were found in only one patient (patient 14). CSF oligoclonal bands were positive in four patients in the GFAP-AQP4 group and two patients in the GFAP-MOG group. Next-generation sequencing (NGS) detected herpes simplex virus in patient 15. In addition, one patient (patient 1) in the GFAP-AQP4 group tested positive for anti-*N*-methyl-d-aspartate receptor (NMDAR) antibody, antiglutamic acid decarboxylase 65 (GAD65) antibody, and antiglycine receptor (GlyR) antibody in the CSF. Two patients (patients 10 and 16) in the GFAP-MOG group tested positive for anti-NMDAR antibody in both CSF and serum. No differences in MRI lesions or CSF findings were observed between the two overlapping syndromes (**Table 3**).

#### 3.1.3 Treatment and outcome

All patients received intravenous methylprednisolone (IVMP; 0.5–1 g/day for 5 days in 14 patients, 0.3 g/day in patient 12, and 0.2 g/day in patient 13) during the acute stage, followed by a tapering course of oral methylprednisolone (decreased by 4 mg/day every 1–2 weeks). Nine patients received intravenous immunoglobulin (IVIG; 0.4 g/kg/day for 5 days), while one patient underwent plasma exchange. Additionally, seven patients in the GFAP-AQP4 group and three patients in the GFAP-MOG group received long-term immunotherapy. Most patients showed good outcomes after treatment.

During follow-up, three patients (patients 1, 4, and 8) in the GFAP-AQP4 group became negative for both GFAP-IgG and AQP4-IgG, with clinical improvement. Patient 3 experienced a recurrence of myelitis 7 months later, accompanied by elevated AQP4 antibody titers. In the GFAP-MOG group, three patients (patients 10, 13, and 15) became negative for both GFAP-IgG and MOG-IgG. Patient 13 experienced a recurrence of myelitis and ON 5 months later, with MOG-IgG reverting to positive. No differences in treatment and outcome were observed between the two overlapping syndromes (**Table 3**).

### 3.2 Comparison of clinical features among different groups

Of the 81 GFAP-IgG-positive patients, 16 patients with AQP4-IgG or MOG-IgG were classified into the overlapping syndrome group, while 65 patients without AQP4-IgG or MOG-IgG were classified into the nonoverlapping syndrome group.

#### 3.2.1 Overlapping syndrome and nonoverlapping syndrome groups

Compared with the nonoverlapping syndrome group, the overlapping syndrome group had a higher proportion of women (68.6% vs. 32.3%, *p* = 0.008), more cases of ON (43.8% vs. 4.6%, *p* < 0.001), lower CSF white blood cell counts (median: 30 cells/mm^3^ vs. 94 cells/mm^3^, *p* = 0.001) and protein levels (median: 0.375 g/L vs. 0.78 g/L, *p* < 0.001). Furthermore, a greater proportion of patients in the overlapping syndrome group received long-term immunotherapy (68.8% vs. 13.8%, *p* < 0.001) (**Table 3**).

#### 3.2.2 GFAP-AQP4 and nonoverlapping syndrome groups

Compared with the nonoverlapping syndrome group, the GFAP-AQP4 group had a higher proportion of women (88.9% vs. 32.3%, *p* = 0.004), lower CSF white blood cell counts (median: 31 cells/mm^3^ vs. 94 cells/mm^3^, *p* = 0.011) and protein levels (median: 0.368 g/L vs. 0.78 g/L, *p* < 0.001), and a greater proportion of patients receiving long-term immunotherapy (77.8% vs. 13.8%, *p* < 0.001) (**Table 3**).

#### 3.2.3 GFAP-MOG and nonoverlapping syndrome groups

Compared with the nonoverlapping syndrome group, the GFAP-MOG group exhibited a higher frequency of ON (71.4% vs. 4.6%, *p* < 0.001) and seizures (57.1% vs. 15.4%, *p* = 0.032), more cortical lesions (85.7% vs. 35.4%, *p* = 0.003), lower CSF white blood cell counts (median: 22 cells/mm^3^ vs. 94 cells/mm^3^, *p* = 0.020) and protein levels (median: 0.381 g/L vs. 0.78 g/L, *p* = 0.004), and a greater proportion of patients receiving long-term immunotherapy (57.1% vs. 13.8%, *p* = 0.021) (**Table 3**).

## 4 Discussion

In recent years, with the expansion of the spectrum of antineuronal antibodies and advances in detection techniques, an increasing number of CNS cases with overlapping antibodies have been reported. Some patients may test positive for multiple neuronal antibodies or present with overlapping clinical phenotypes. Previous studies have shown that GFAP-IgG can coexist with other neuronal antibodies, most commonly NMDAR-IgG, AQP4-IgG, and MOG-IgG. However, the prevalence of coexisting antibodies varies considerably across different studies (1, 6, 11–13). In a report from the Mayo Clinic, Flanagan et al. found that among 102 A-GFAP-A patients, 41 (40%) had one or more coexisting antibodies. The most common was NMDAR-IgG, followed by AQP4-IgG (1). Yang et al. analyzed 30 GFAP-IgG-positive patients, of whom 10 (33.3%) were diagnosed with overlapping syndromes. AQP4-IgG was the most common coexisting antibody (16.6%), followed by NMDAR-IgG (6). However, some scholars have suggested that MOG-IgG is the most frequently coexisting antibody. A Chinese study of 35 children with A-GFAP-A showed that 11 tested positive for other neuronal antibodies, including MOG-IgG in five patients (14.3%) (11). Fang et al. also reported that MOG antibody coexistence was the most common type of A-GFAP-A overlapping syndrome, with an occurrence rate of 10.4% (12).

In our study, the clinical characteristics of these two overlapping syndromes can be summarized as follows: (1) In our cohort, the prevalence of GFAP-IgG coexisting with AQP4-IgG and MOG-IgG was comparable. (2) Symptoms of the overlapping syndromes were diverse, exhibiting features consistent with both A-GFAP-A and the diagnostic criteria for NMOSD or MOGAD. (3) Patients with GFAP-IgG coexisting with AQP4-IgG or MOG-IgG were more likely to be women, exhibited a higher prevalence of ON, and had milder CSF inflammatory changes. (4) Antibody detection is crucial for disease diagnosis; however, antibody levels may fluctuate during disease progression and in response to treatment.

Our study found a higher proportion of women in the GFAP-AQP4 group, consistent with the predominance of women observed in NMOSD. This may be due to the greater likelihood of AQP4 antibodies affecting women. Yang et al. reported that patients with overlapping syndromes tended to be younger (6); however, this age-related difference was not observed in our study. Although the clinical manifestations of overlapping syndromes are diverse and difficult to distinguish from those of nonoverlapping syndromes, several distinguishing features can still be identified. In our study, patients with overlapping syndromes exhibited a significantly higher prevalence of ON. Particularly in the GFAP-MOG group, ON was the most common phenotype. Patients with overlapping syndromes may present with bilateral ON or recurrent ON, with relapses predominantly occurring during steroid tapering. In addition, MRI revealed T2-hyperintensities in the optic nerves or optic chiasm, consistent with ON features observed in demyelinating diseases (3, 4, 9). In contrast, visual impairment in A-GFAP-A is heterogeneous, and optic disk edema is more likely attributable to venous inflammation rather than ON (12, 14, 15). NMOSD usually affects the optic nerves and spinal cord, with less involvement of the brain (16). However, in our cohort, 77.8% of patients in the GFAP-AQP4 group exhibited symptoms of encephalopathy. This discrepancy may be attributed to the coexistence of GFAP-IgG. APS is a characteristic manifestation in AQP4-IgG-positive NMOSD, with a reported prevalence of 16% to 43% (3). It can also occur in A-GFAP-A, albeit at a lower frequency (4%–11%) (17, 18). In our GFAP-AQP4 group, two patients experienced APS, similar to previous studies (19). Patients with MOGAD were more likely to experience seizures than those with NMOSD or A-GFAP-A (5, 20). Similarly, we observed a higher incidence of seizures in the GFAP-MOG group, with MRI revealing more cortical involvement. Therefore, seizures may serve as a potential indicator of MOG antibody positivity. These findings suggest that overlapping phenotypes may be driven by overlapping autoimmune mechanisms.

Intracranial lesions in A-GFAP-A predominantly involve the cerebral white matter, basal ganglia, hypothalamus, cerebellum, and brainstem. Periventricular radial linear enhancement and leptomeningeal enhancement are characteristic imaging features of the disease (1, 2, 5, 9). In our cohort, three patients in the overlapping syndrome group exhibited leptomeningeal enhancement, and five patients presented with clinical signs of meningeal irritation. These findings suggest a possible role for GFAP-IgG in these conditions. Moreover, periventricular lesions commonly seen in NMOSD and large space-occupying lesions typically associated with MOGAD can also be present in patients with overlapping syndromes. Patients with NMOSD and MOGAD often present with mild CSF pleocytosis and elevated protein levels, whereas those with A-GFAP-A exhibit a more severe inflammatory response (5, 16). In our study, the CSF of patients with overlapping syndromes exhibited mild inflammatory changes, closely resembling those observed in demyelinating diseases. These findings may reflect the pathogenic effects of AQP4-IgG or MOG-IgG.

Infection is a common immune trigger of antibody-mediated autoimmune disorders (21, 22). These pathogens may directly damage neuronal and glial cells, leading to antibody production and secondary autoimmune responses. In addition, certain components of these pathogens may share structural similarities with host antigens, increasing the risk of generating multiple antibodies. Unlike AQP4 or MOG, which target cell surface antigens and exert direct pathogenic effects, GFAP is an intracellular antigen. Rather than acting as a direct pathogenic target, GFAP serves as a biomarker of CD8+ T-cell-mediated inflammatory processes (1, 2). Autopsy findings from a GFAP-IgG-positive patient with meningoencephalomyelitis were nonspecific and showed no astrocyte involvement or demyelination (23), suggesting that GFAP-IgG may not be the directly pathogenic antibody responsible for astrocyte inflammation. AQP4-IgG or MOG-IgG from the systemic circulation may enter the CNS through a disrupted blood–brain barrier, potentially initiating primary inflammatory events and damaging astrocytes, while GFAP autoimmunity may occur as a secondary phenomenon. Elevated levels of GFAP have been shown to correlate with disease severity in AQP4-IgG-positive NMOSD and MOGAD, particularly during acute attacks (24, 25). Moreover, the presence of multiple antibody positivities may also be related to immune reconstitution (26). During the reduction or cessation of immunotherapy, the immune system recovers from immunosuppression and rebuilds itself, leading immune cells to attack autoantigens and generate new immune responses (27, 28). However, the precise mechanisms underlying overlapping syndromes remain poorly understood, and further research is warranted.

In addition to AQP4-IgG and MOG-IgG, GFAP-IgG can coexist with other antibodies, most commonly NMDAR-IgG (1). Interestingly, three patients with overlapping syndromes tested positive for NMDAR-IgG, including two in the GFAP-AQP4 group and one in the GFAP-MOG group. MOG and functional NMDARs may coexist on the surface of oligodendrocytes. During autoimmune processes, immune cells may mistakenly target MOG and NMDAR autoantigens, leading to the production of MOG-IgG and NMDAR-IgG (26, 29). The co-occurrence of GFAP-IgG, AQP4-IgG, and NMDAR-IgG is a strong predictor of ovarian teratoma. Flanagan et al. reported that among seven GFAP-IgG-positive patients who were concurrently positive for NMDAR-IgG and AQP4-IgG, five developed ovarian teratoma (1). GFAP autoimmunity may represent a paraneoplastic immune response triggered by tumors expressing neuronal and glial elements. However, no tumors were observed in our study of overlapping syndromes, possibly due to the limited sample size or short follow-up period. Additionally, some scholars have suggested that the incidence of tumors may be associated with ethnic specificity (30).

The diagnosis of overlapping syndrome primarily relies on antibody detection. For A-GFAP-A, CSF antibody testing demonstrates high specificity and sensitivity. However, the phenotypes of patients with positive serology are heterogeneous (1, 31–34). Therefore, patients with isolated serum GFAP-IgG positivity require additional clinical and radiological assessments. In our study, two patients with overlapping syndromes tested positive for GFAP-IgG only in the serum. Nevertheless, after a rigorous exclusion of other neurological disorders, both cases fulfilled the diagnostic criteria for A-GFAP-A. It should be noted that antibody testing alone is insufficient to establish a definitive diagnosis, as false-positive results may occur. Therefore, we recommend repeating antibody testing, particularly during disease relapses.

Although antibody overlap can complicate clinical diagnosis, the management of overlapping and nonoverlapping syndromes during the acute phase follows similar therapeutic principles. Patients with A-GFAP-A overlapping syndromes typically respond poorly to acute-phase therapy and exhibit higher relapse rates. Consequently, aggressive immunotherapy is recommended for these patients (6, 9, 11).

Here, we provide the following recommendations: (1) For patients who test positive for AQP4 or MOG antibodies, testing for GFAP antibodies is not recommended, as the presence of AQP4 or MOG antibodies is sufficient to guide subsequent treatment decisions. (2) For patients who test positive for GFAP antibodies, it is recommended to simultaneously test for AQP4 and MOG antibodies. Given the recurrent and disabling nature of NMOSD and MOGAD, long-term immunotherapy is of great significance. Detection of AQP4 and MOG antibodies may aid in informing subsequent treatment strategies. (3) If patients with encephalitis or myelitis develop ON either simultaneously or sequentially, it is recommended to test for AQP4 or MOG antibodies first, rather than GFAP antibodies.

This study has several limitations. First, as a retrospective study, it limits the collection of detailed clinical data, such as antibody titers, the possible presence of other unknown pathogenic antibodies, and comprehensive ophthalmological assessments. Second, the use of a fixed CBA for antibody detection may result in false-negative results, particularly in low-titer cases. Some patients did not undergo antibody testing during the recovery phase. Additionally, due to the small sample size and multiple statistical comparisons, there is a risk of type I error. Given the retrospective and exploratory nature of the study, *p*-values should not be interpreted with caution and should not be considered confirmatory.

In conclusion, A-GFAP-A can coexist with demyelinating diseases. When patients with A-GFAP-A present with atypical symptoms such as ON, or when patients with demyelinating diseases have meningeal involvement, the possibility of an overlapping syndrome should be considered. Accurate diagnosis requires both clinical assessment and antibody testing. Future research should aim to elucidate the association between antibodies and clinical phenotypes, identify pathogenic antibodies, and avoid relying solely on antibody presence for diagnosis.

## Data Availability

The raw data supporting the conclusions of this article will be made available by the authors, without undue reservation.

## References

[1] Fang B McKeon A Hinson SR Kryzer TJ Pittock SJ Aksamit AJ. Autoimmune glial fibrillary acidic protein astrocytopathy: a novel meningoencephalomyelitis. JAMA Neurol. (2016) 73:1297–307. doi: 10.1001/jamaneurol.2016.2549, PMID: 27618707

[2] Flanagan EP Hinson SR Lennon VA Fang B Aksamit AJ Morris PP. Glial fibrillary acidic protein immunoglobulin G as biomarker of autoimmune astrocytopathy: analysis of 102 patients. Ann Neurol. (2017) 81:298–309. doi: 10.1002/ana.24881, PMID: 28120349

[3] Wingerchuk DM Banwell B Bennett JL Cabre P Carroll W Chitnis T. International consensus diagnostic criteria for neuromyelitis optica spectrum disorders. Neurology. (2015) 85:177–89. doi: 10.1212/WNL.0000000000001729, PMID: 26092914 PMC4515040

[4] Banwell B Bennett JL Marignier R Kim HJ Brilot F Flanagan EP. Diagnosis of myelin oligodendrocyte glycoprotein antibody-associated disease: international MOGAD Panel proposed criteria. Lancet Neurol. (2023) 22:268–82. doi: 10.1016/S1474-4422(22)00431-8, PMID: 36706773

[5] Xiao J Zhang S Chen X Tang Y Chen M Shang K. Comparison of clinical and radiological characteristics in autoimmune GFAP astrocytopathy, MOGAD and AQP4-IgG^+^ NMOSD mimicking intracranial infection as the initial manifestation. Mult Scler Relat Disord. (2022) 66:104057. doi: 10.1016/j.msard.2022.104057, PMID: 35870369

[6] Yang X Xu H Ding M Huang Q Chen B Yang H.Overlapping autoimmune syndromes in patients with glial fibrillary acidic protein antibodies Front Neurol. (2018) 9:251. doi: 10.3389/fneur.2018.00251, PMID: 29755396 PMC5932346

[7] Martin AJ Strathdee J Wolfe N. Coexistent anti-GFAP and anti-MOG antibodies presenting with isolated meningitis and papillitis: more support for overlapping pathophysiology. BMJ Neurol Open. (2022) 4:e000236. doi: 10.1136/bmjno-2021-000236, PMID: 35402916 PMC8948407

[8] Zhao C Li A Liu L Wang J Fan D. Acute myelitis, recurrent optic neuritis, and seizures over 17 years. Front Neurol. (2020) 11:541146. doi: 10.3389/fneur.2020.541146, PMID: 33281696 PMC7691272

[9] Ding J Ren K Wu J Li H Sun T Yan Y. Overlapping syndrome of MOG-IgG-associated disease and autoimmune GFAP astrocytopathy. J Neurol. (2020) 267:2589–93. doi: 10.1007/s00415-020-09869-2, PMID: 32378036

[10] Ji S Liu C Bi Z Gao H Sun J Bu B. Overlapping syndrome mimicking infectious meningoencephalitis in a patient with MOG and GFAP IgG. BMC Neurol. (2021) 21:348. doi: 10.1186/s12883-021-02381-8, PMID: 34507542 PMC8431933

[11] Fang H Hu W Jiang Z Yang H Liao H Yang L. Autoimmune glial fibrillary acidic protein astrocytopathy in children: a retrospective analysis of 35 cases. Front Immunol. (2021) 12:761354. doi: 10.3389/fimmu.2021.761354, PMID: 34880859 PMC8645641

[12] Fang T Wu W He X Liang Y Lin Q Dai K. Clinical characteristics of overlapping syndrome in patients with GFAP-IgG and MOG-IgG: a case series of 8 patients and literature review. J Neurol. (2024) 271:6811–21. doi: 10.1007/s00415-024-12633-5, PMID: 39190107

[13] Liu L Fang B Qiao Z Di X Ma Q Zhang J. Clinical manifestation, auxiliary examination features, and prognosis of GFAP autoimmunity: a Chinese cohort study. Brain Sci. (2022) 12:1662. doi: 10.3390/brainsci12121662, PMID: 36552122 PMC9775969

[14] Chen JJ Aksamit AJ McKeon A Pittock SJ Weinshenker BG Leavitt JA. Optic disc edema in glial fibrillary acidic protein autoantibody-positive meningoencephalitis. J Neuroophthalmol. (2018) 38:276–81. doi: 10.1097/WNO.0000000000000593, PMID: 29210929

[15] Greco G Masciocchi S Diamanti L Bini P Vegezzi E Marchioni E. Visual system involvement in glial fibrillary acidic protein astrocytopathy: two case reports and a systematic literature review. Neurol Neuroimmunol Neuroinflamm. (2023) 10:e200146. doi: 10.1212/NXI.0000000000200146, PMID: 37582612 PMC10427126

[16] Lana-Peixoto MA Talim N. Neuromyelitis optica spectrum disorder and anti-MOG syndromes. Biomedicines. (2019) 7:42. doi: 10.3390/biomedicines7020042, PMID: 31212763 PMC6631227

[17] Deng B Wang J Yu H Jin L Qiu Y Liu X. Area postrema syndrome in autoimmune glial fibrillary acidic protein astrocytopathy: a case series and literature review. Neurol Neuroimmunol Neuroinflamm. (2022) 9:e200029. doi: 10.1212/NXI.0000000000200029, PMID: 36163176 PMC9513980

[18] Long Y Liang J Xu H Huang J Yang J Gao C. Autoimmune glial fibrillary acidic protein astrocytopathy in Chinese patients: a retrospective study. Eur J Neuol. (2018) 25:477–83. doi: 10.1111/ene.13531, PMID: 29193473

[19] Lin H Huang Y Zeng H Wang M Guan S Chen G. Overlapping clinical syndromes in patients with glial fibrillary acidic protein IgG. Neuroimmunomodulation. (2020) 27:69–74. doi: 10.1159/000505730, PMID: 32101879

[20] Hamid SHM Whittam D Saviour M Alorainy A Mutch K Linaker S. Seizures and encephalitis in myelin oligodendrocyte glycoprotein IgG disease vs aquaporin 4 IgG disease. JAMA Neurol. (2018) 75(1):65–71. doi: 10.1001/jamaneurol.2017.3196, PMID: 29131884 PMC5833490

[21] Koga M Takahashi T Kawai M Fujihara K Kanda T. A serological analysis of viral and bacterial infections associated with neuromyelitis optica. J Neurol Sci. (2011)300(1-2):19–22. doi: 10.1016/j.jns.2010.10.013, PMID: 21056429

[22] Frau J Coghe G Lorefice L Fenu G Cocco E. The Role of microorganisms in the etiopathogenesis of demyelinating diseases. Life (Basel). (2023) 13:1309. doi: 10.3390/life13061309, PMID: 37374092 PMC10305018

[23] Yamakawa M Hogan KO Leever J Jassam YN. Autopsy case of meningoencephalomyelitis associated with glial fibrillary acidic protein antibody. Neurol Neuroimmunol Neuroinflamm. (2021) 8:e1081. doi: 10.1212/NXI.0000000000001081, PMID: 34642236 PMC8515200

[24] Chang X Huang W Wang L Zhang J Zhou L Lu C. Serum neurofilament light and GFAP are associated with disease severity in inflammatory disorders with aquaporin-4 or myelin oligodendrocyte glycoprotein antibodies. Front Immunol. (2021) 12:647618. doi: 10.3389/fimmu.2021.647618, PMID: 33796113 PMC8008082

[25] Schindler P Aktas O Ringelstein M Wildemann B Jarius S Paul F. Glial fibrillary acidic protein as a biomarker in neuromyelitis optica spectrum disorder: a current review. Expert Rev Clin Immunol. (2023) 19:71–91. doi: 10.1080/1744666X.2023.2148657, PMID: 36378751

[26] Ren Y Chen X He Q Wang R Lu W. Co-occurrence of anti-N-methyl-D-aspartate receptor encephalitis and anti-myelin oligodendrocyte glycoprotein inflammatory demyelinating diseases: a clinical phenomenon to be taken seriously. Front Neurol. (2019) 10:1271. doi: 10.3389/fneur.2019.01271, PMID: 31866928 PMC6904358

[27] Van Obberghen EK Cohen M Rocher F Lebrun-Frenay C. Multiple immune disorders after natalizumab discontinuation: after the CIRIS, the SIRIS? Rev Neurol (Paris). (2017) 173:222–4. doi: 10.1016/j.neurol.2017.03.008, PMID: 28372806

[28] Giedraitienė N Kizlaitienė R Kaubrys G. New autoimmune disorder development after immune reconstitution therapy for multiple sclerosis. Sci Rep. (2024) 14:30991. doi: 10.1038/s41598-024-82196-y, PMID: 39730657 PMC11681027

[29] Fan S Xu Y Ren H Guan H Feng F Gao X. Comparison of myelin oligodendrocyte glycoprotein (MOG)-antibody disease and AQP4-IgG-positive neuromyelitis optica spectrum disorder (NMOSD) when they co-exist with anti-NMDA (N-methyl-D-aspartate) receptor encephalitis. Mult Scler Relat Disord. (2018) 20:144–52. doi: 10.1016/j.msard.2018.01.007, PMID: 29414288

[30] Zhu B Sun M Yang T Yu H Wang L. Clinical, imaging features and outcomes of patients with anti-GFAP antibodies: a retrospective study. Front Immunol. (2023) 14:1106490. doi: 10.3389/fimmu.2023.1106490, PMID: 37205100 PMC10187143

[31] Zhang Z Zoltewicz JS Mondello S Newsom KJ Yang Z Yang B.Human traumatic brain injury induces autoantibody response against glial fibrillary acidic protein and its breakdown products. PLoS One. (2014)9, e92698. doi: 10.1371/journal.pone.0092698, PMID: 24667434 PMC3965455

[32] Wang KKW Yang Z Yue JK Zhang Z Winkler EA Puccio AM. Plasma anti-glial fibrillary acidic protein autoantibody levels during the acute and chronic phases of traumatic brain injury: a transforming research and clinical knowledge in traumatic brain injury pilot study. J Neurotrauma (2016) 33(13):1270–7. doi: 10.1089/neu.2015.3881, PMID: 26560343 PMC4931336

[33] Wei P Zhang W Yang L Zhang H Xu X Jiang Y. Serum GFAP autoantibody as an ELISA-detectable glioma marker. Tumour Biol. (2013) 34:2283–92. doi: 10.1007/s13277-013-0770-7, PMID: 23589055

[34] Gómez-Touriño I Camiña-Darriba F Otero-Romero I Rodríguez MA Hernández-Fernández A González-Fernández A. Autoantibodies to glial fibrillary acid protein and S100beta in diabetic patients. Diabetes Med. (2010) 27:246–8. doi: 10.1111/j.1464-5491.2009.02911.x, PMID: 20546276

